# Enablers and inhibitors of community participation in Ghana’s Community-based Health Planning and Services programme: a qualitative study in the Builsa North Municipality

**DOI:** 10.1186/s12913-022-08869-4

**Published:** 2022-12-02

**Authors:** Shieghard Agalga, Kennedy A. Alatinga, Gilbert Abotisem Abiiro

**Affiliations:** 1 Department of Community Development, Faculty of Sustainable Development Studies, University for Development Studies, P.O. Box TL 1350, Tamale, Ghana; 2 Department of Community Development, Faculty of Planning and Land Management, SDD- University of Business and Integrated Development Studies, Wa, Ghana; 3 Department of Health Services, Policy, Planning, Management and Economics, School of Public Health, University for Development Studies, Tamale, Ghana; 4 Department of Population and Reproductive Health, School of Public Health, University for Development Studies, Tamale, Ghana

**Keywords:** Primary health care, Community participation, Community-based Health Planning and Services, Ghana

## Abstract

**Background:**

Community participation is essential for the successful implementation of primary health care programmes across the globe, including sub-Saharan Africa. The Community-based Health Planning and Services (CHPS) programme is one of the primary health care interventions in Ghana which by design and implementation heavily relies on community participation. However, there is little evidence to establish the factors enabling or inhibiting community participation in the Ghanaian CHPS programme. This study, therefore, explored the enabling and inhibiting factors influencing community participation in the design and implementation of the CHPS programme in the Builsa North Municipality in the Upper East Region of Ghana.

**Methods:**

A qualitative approach, using a cross-sectional design, was employed to allow for a detailed in-depth exploration of the enabling and inhibiting factors influencing community participation in the design and implementation of the CHPS programme. The data were collected in January 2020, through key informant interviews with a stratified purposive sample of 106 respondents, selected from the 15 functional CHPS facilities in the Municipality. The data were audio-recorded, transcribed and manually analysed using thematic analysis.

**Results:**

The results showed that, public education on the CHPS concept, capacity of the community to contribute material resources towards the construction of CHPS facilities, strong and effective community leadership provided by community chiefs and assembly persons, the spirit of volunteerism and trust in the benefits of the CHPS programme were the enablers of community participation in the programme. However, volunteer attrition, competing economic activities, lack of sense of ownership by distant beneficiaries, external contracting of the construction of CHPS facilities and illiteracy constituted the inhibiting factors of community participation in the programme.

**Conclusion:**

Extensive public education, volunteer incentivization and motivation, and the empowerment of communities to construct their own CHPS compounds are issues that require immediate policy attention to enhance effective community participation in the programme.

## Background

Inequitable distribution of health services has been a major issue across the globe, especially in Sub-Saharan Africa [1–3]. This has resulted in a healthcare access gap between the rich and the poor, which necessitated the Alma Ata declaration and the subsequent adoption of primary health care (PHC) by the World Health Organization (WHO) and its member states in 1978 [4]. WHO and the United Nations Children’s Fund (UNICEF) defined PHC as “essential health care based on practical, scientifically sound and socially acceptable methods and technology made universally accessible to individuals and families in the community through their full participation and at a cost that the community and country can afford to maintain at every stage of their development in the spirit of self-reliance and self-determination” [5]. According to WHO [6], PHC focuses on addressing the main health problems of the vulnerable and the excluded in the community in order to address the equity gaps in health service delivery.

With the adoption of PHC as the official health policy direction for the member states of WHO, community participation assumed an important role in global health policy [7–9]. According to Oakley and WHO [10], “community participation is a process by which partnership is established between the government and local communities in the planning, implementation and utilization of health activities in order to benefit from self-reliance and social control over the infrastructure and technology of primary health care”. As a concept, community participation has increasingly been recognized as key to implementing and sustaining interventions that improve health outcomes [11–13]. The benefits of community participation include the fact that it leads to more responsive care, facilitates people’s involvement in treatment decisions, and improves quality and safety of care [14].

Since the recognition of the role of community participation in PHC by WHO, many countries across the globe, including sub-Saharan African countries, have been implementing programmes that focus on addressing the health needs of the people, especially the most deprived and marginalized through their active participation [11, 15]. In Ghana, one of such programmes is the Community-based Health Planning and Services programme (CHPS) [9, 16]. The CHPS programme was introduced in 1999 to demonstrate a paradigm shift from the traditional approach to health care delivery which was characterized by bureaucratic models of service delivery [7, 17]. Its overall goal is to increase access to rural health care services while at the same time empowering communities to take greater control over their health by actively participating in the implementation of PHC services and activities through the mobilization of community leadership, decision making systems and resources within the coverage area of community leaders and members [16]. In effect, community participation serves as the chief corner stone for the successful implementation of the CHPS programme [18].

The design of the Ghanaian CHPS programme requires the participation of beneficiary communities in the planning and implementation of key components of the programme such as needs assessment, leadership, organization, resource mobilization and management, in line with the five dimensions of the Rifkin’s model of participation in health programmes [7, 19]. Wright et al. [20], suggest that the identification of the needs of the community with community members is very critical in the planning and implementation of local health services. Rifkin et al. [19] states that in measuring community participation in the planning and implementation of health care activities, it is imperative to examine who the existing leadership represents, how the leadership acts on the interest of various community groups, especially the poor, and how responsive the leaders are to change. The organization dimension refers to the extent to which new health programmes are integrated within pre-existing community structures or networks [7]. Resource mobilization refers to the capacity of communities to galvanize and contribute the relevant resources towards the successful implementation of community–based health interventions [19]. Community participation in resource mobilization is critical to the ownership and sustainability of any project as it serves as a condition for breaking the shackles of dependency and passivity [21]. The management dimension refers to the capacity of the beneficiary community to take absolute control over the decision-making process about the programme’s development and implementation [7]. However, the successes of community participation in the various dimensions of a health programme as outlined by Rifkin depend on the influences of both enabling and inhibiting factors [19].

According to the CHPS operational policy document, 2016, communities are required to play a key role in the identification and prioritization of their health needs, as well as assisting Community Health Officers in the recruitment of volunteers [16]. These volunteers are supposed to be responsible for delivering basic health support services and essential medicines to households as well as taking decisions on behalf of the entire community with regards to the management of the programme [16]. Communities are further required to mobilize financial, material and human resources towards the construction and maintenance of the CHPS facility. In terms of organization, communities are expected to create their own mechanisms for introducing health programmes.

However, in spite of the significance of community participation in the success of PHC programmes such as the CHPS initiative, the extent to which communities participate in the planning and implementation of PHC programmes is still questioned [7, 17, 22]. Despite a scale-up of the CHPS programme across the country, the programme continues to be managed by biomedical scientists and health professionals whose technocratic solutions to ill health create few opportunities for community members to be able to apply these solutions to local realities [7]. Even though contemporary scholars have been advocating for the inclusion of people participation in the CHPS programme, much of these works focus on establishing the link between community participation and health outcomes [3, 7, 9, 14, 17, 23]. There is little evidence to establish the enabling and inhibiting factors influencing community participation in the CHPS programme. This paper, therefore, seeks to explore the enabling and inhibiting factors influencing community participation in the design and implementation of the CHPS programme in Ghana.

## Methods

### Study setting

The study was conducted in the Builsa North Municipality in the Upper East Region of Ghana. The Builsa North Municipality was purposively chosen for the study because, it is predominantly rural with limited accessibility to higher level health care facilities such as hospitals and health centres [24]. The residents of the Municipality, therefore, largely depend on CHPS facilities for health care [24]. The municipality had a total population of 56,477 constituting 5.4% of the region’s population and 0.2% of the country’s total population in 2010 [25]. Females constitute 50.8% [25]. The municipality is made up of 98 communities clustered into five town/ area councils namely Chuchuliga, Kadema, Sandema, Siniensi and Wiaga. About 90% of the population is rural [25]. The municipality has one hospital situated in its capital (Sandema), three health centres established in Chuchuliga, Wiaga, and Siniensi. The hospital serves as the referral facility that links up with these smaller health centres and the community level health facilities. Fifteen CHPS compounds have been established so far across the municipality to provide community-based health care services. The literate population of the municipality constitutes 50.5% [25]. Majority of the populace representing 83.1% of households in the municipality are engaged in agriculture [25]. Crop farming is the main agricultural activity with 96.3% of households engaged in it [25]. The settlement pattern of the municipality is highly dispersed, most especially in the rural communities as houses/residential structures are widely apart.

### Study design and sampling

A qualitative approach, using a cross-sectional design, was employed to allow for a detailed in-depth exploration of the enabling and inhibiting factors influencing community participation in the design and implementation of the CHPS programme. The study targeted key stakeholders of the CHPS programme in the municipality. A stratified purposive sampling technique was employed to select 106 respondents across all the 15 CHPS facilities in the municipality. These respondents included 15 divisional chiefs, 15 assembly persons, 15 *”mangazias”* [women spokesperson], 15 chairpersons of health committee, 15 health volunteer chairpersons, 15 chairpersons of the mother-to-mother health support groups, 15 midwives and the Community Engagement Officer of the Municipal Health Directorate. The rationale for purposefully selecting this category of persons as respondents include the following; divisional chiefs are heads of the traditional political system and serve as the traditional mouth piece of the communities as well as the first point of contact before CHPS implementation. In addition, unlike the divisional chiefs, assembly persons are the heads of the modern political system who serve as the political mouth piece of the communities and also the first point of contact together with the chiefs before CHPS implementation. Furthermore, the chairperson of the health committee is the head of the health committee that is responsible for the implementation of community decisions on the management of CHPS. Moreover, the chairperson of the health volunteers is the head of the health volunteers who are responsible for educating individuals and households on basic health issues and also serve as agents of referral services and community mobilization. The chairperson of the mother-to-mother health group is also responsible for supervising the dissemination of health-related information to women. “*Magazias”* represent the voice of women in the communities. Midwives are responsible for managing the CHPS facilities, supervising the recruitment of health volunteers and mother-to-mother health support groups as well as building their capacities. The Community Engagement Officer at the Municipal Health Directorate is also responsible for engaging beneficiary communities in the implementation process of the CHPS programme. In recruiting the study participants, especially chairpersons of health committee, volunteers and mother-to-mother health groups, we obtained a list containing their names from the 15 CHPS facilities and then contacted them with the assistance of the assembly persons and chiefs.

### Data collection and analysis

Data were collected from respondents using key informant interviews (KIIs). We developed a KII guide which contained open-ended questions and probes about community participation in needs assessment, leadership, organization, resource mobilization, and management of CHPS as well as the factors that enable and inhibit community participation in the programme. The interviews were carried out in January 2020 by three trained research assistants and under the supervision of the first author. The interviews were conducted at the private residence of the respondents and in English or Buli (local language) depending on the preference of the interviewee. Each interview lasted between 45 and 60 min. The interviews were audio-recorded under consent and later transcribed. The interviews that were conducted in Buli were back translated into English during the transcription. This was done for the purpose of enhancing consistency in the representation of the views of respondents.

The interview guide was piloted 3 months before the commencement of the study. The study also employed data triangulation by relying on multiple sources of data. The data were sourced from staff of the Builsa North District Health Directorate, health committee members, volunteers, community leaders and mother-to-mother women groups. Member checking was also done by ensuring that each audio recording was played in the presence of the respondent for validation of the responses. All methods were implemented in line with the Principles of the Declaration of Helsinki on ethical principles for medical research involving human subjects [26].

Thematic analysis was employed to analyze the data on the enablers and inhibitors of community participation in the planning and implementation of the CHPS programme. The data were analyzed manually. Since we did not adopt a clear theoretical/conceptual framework to guide the study, the development of themes during the analysis was data driven and carried out in tandem with inductive coding of the data by listing down phrases that captured emerging concepts [27]. The inductive approach to coding allowed for important non-preconceived themes, reflecting salient research findings, to emerge from the frequent issues inherent in the raw data. SA and GAA independently coded a sample of 20 transcripts simultaneously. The two authors discussed the outcome of their independent coding to identify emerging common themes as a framework to guide the subsequent coding of the remaining transcripts. Guided by these preliminary themes, SA coded the rest of the transcripts and the results were reviewed by KAA and GAA. All themes derived from the inductive coding were re-categorized into enablers and inhibitors of participation in line with the objective of the study. The findings are presented with the support of direct quotations from the transcripts.

## Results

### Summary of results

The enablers of community participation identified from the thematic analysis were: public education on the CHPS initiative, capacity to contribute material resources towards the construction of CHPS compounds, strong and effective community leadership provided by community chiefs and assembly persons, spirit of volunteerism, and trust in the benefits of the CHPS programme. On the other hand, the inhibitors of participation were identified as: volunteer attrition, competing economic activities, lack of sense of ownership of CHPS by distant beneficiaries, external contracting of the construction of CHPS facilities and illiteracy. Each of these enablers and inhibitors of community participation in the programme are further described in the subsequent subsections.

### Enablers of community participation in CHPS

#### Public education on the CHPS initiative

The results showed that, awareness of the existence of the CHPS initiative, created through public education, is a key enabler of community participation. Majority of respondents across the various zones reported that, beneficiary communities who clearly understood the CHPS concept and its operations were motivated to play leading roles in supporting its planning and management.

*“We were able to hold a couple of meetings as a community to plan and discuss how the CHPS programme was going to be implemented. This was made possible after we were informed about the programme and our role in its implementation by the health team” *(Key Informant 18, Chuchuliga Zone, Male)

*“Before we were involved in the planning and implementation of the CHPS programme, the District Health Management Team came and held a meeting with us concerning the introduction of the programme in the community and the role we were supposed to play in its establishment.”* (Key Informant 105, Wiaga Zone, Female)

### Capacity to contribute material resources towards the construction of CHPS facilities

Responses gathered indicated that, the communities participated in the construction and maintenance of their CHPS facilities because they were able to contribute material resources, such as land, labour, and building materials, towards the construction and maintenance of the CHPS compounds.

*“Our participation in this initiative was motivated by our ability to contribute the resources (labour and building materials) that were required for the construction and maintenance of the compound. Otherwise, we would have been sitting and watching or not taking key interest in the construction process since we could not offer any support.”* (Key Informant 28, Kadema Zone, Male)

*“We would have had no interest in this initiative, if we were unable to support with the needed resources in raising this facility.” (Key Informant 44, Sandema Zone, Female)*

*“In all the communities where CHPS compounds were constructed, community members were very helpful in mobilizing resources to support the process. For example at Bagyangsa in Kadema the community provided land, they fetched water, stones, molded blocks, and provided some iron rods to support the raising of the compound.”(Key Informant 1, Community Engagement Officer)*

### Strong and effective community leadership provided by community chiefs and assembly persons

Findings from the in-depth interviews also suggested that communities were able to participate in the planning and implementation of the CHPS programme due to strong and effective leadership provided by their chiefs and assembly persons. It was revealed that the mobilization of the communities to discuss and take steps to solve their health problems and the writing of proposals and follow ups predating the establishment of the programme were facilitated by leaders of the various communities. It was further established from the interviews that the leadership of the communities played an active role in the mobilization of labour and other resources when it came to the construction of the health compounds by way of talking to community members to support.

*“The assembly man and the sub chief called us and spoke to us to all contribute in every way to help in the construction of the facility and because we have respect for them we obeyed. So every household contributed money, the women fetched water, the men fetched sand, stones, molded blocks and we all participated in putting up the building.”* (Key Informant 84, Siniensi Zone, Male).

*“Our sub chief, the assembly man together with other opinion leaders have been very active and it is because of their hard work that is why we now have a CHPS compound built in this community.” (Key Informant 14, Chuchuliga Zone, Female).*

### The spirit of volunteerism

The results indicated that members of the various communities willingly offered their services to the programme without any monetary reward in return. For example, the communities voluntarily contributed cash, labour, and building materials without any coercion or monetary enticement to support the construction of the CHPS compounds. Also, the services run by the health committees, volunteers and mother-to-mother support groups are free of charge.

*“All that we are doing to support the CHPS programme is for free, we are not paid for our services but because we love our community, we have no choice. We have been sacrificing our time, resources and energy to support this programme” (Key Informant 31, Kadema Zone, Female).*

*“Each time we are called, we abandon everything of ours including what will bring us money to respond. We do everything they ask us to do, and we offer ourselves to be trained to help the programme. Even the building of the CHPS compound, they asked as to contribute money, we fetched water, stones, sand and molded blocks. We even helped in the building and roofing of the place without any pay.” (Key Informant 93, Wiaga Zone, Male).*

*“In fact, I must commend the efforts of the communities in supporting the programme. They are doing very well, but for them I could not see how this programme was going to succeed. The health committees, volunteers and mother-to-mother support groups have all been working to support the programme without any salary from government or the Ministry of Health.” (Key Informant 1, Community Engagement Officer*)

### Trust in the benefits of the CHPS programme

Trust in the benefits of the CHPS programme was described by participants as one of the enablers of community participation in the planning and implementation of the programme. Members of the various communities had hopes that the programme was going to address their health needs and hence decided to fully participate in its activities.

*“You know in everything if there is no trust you cannot achieve or do anything. We trusted that the programme was going to resolve our health problems and so whatever we were asked to do we did exactly that in anticipation of good results.”* (Key Informant *72, Siniensi Zone, Sub Chief*)

*“So because of the trust we had in the programme, we gave out land, we contributed resources both in kind and in cash to build the health compound and we are also helping the nurses to disseminate information to households in the community concerning health and what have you.”* (Key Informant 86, Wiaga Zone, Male)

### Inhibitors of community participation in CHPS

#### Volunteer attrition

The views shared by participants showed that, most of the health volunteers who have been recruited for the programme, abandoned the work at some point in time due to non-payment and migrated to the southern part of the country in search for greener pastures. This, they indicated negatively affects community participation in the programme.

*“Because we are not being paid for whatever we are doing, after working for some time most of us stop the work and go to the south to look for work that will fetch us money especially during the dry season.”* (Key Informant 42, Kadema Zone, Male)

*“This work we are doing is for free, they do not pay us. There are times you want money to buy ingredients to prepare food for your family and it is difficult. Because of that, after working for some time we leave the work and relocate to the south to look for money and return.”*(Key Informant 78, Siniensi, Male)

*“The volunteers that we normally train to support the programme, they will be in the system for a while because, there is no income from the work they are doing they would like to move down south to look for greener pastures. So, we train and then they leave the system and we have to train again.”*(Key Informant 10, Chuchuliga Zone, Female)

### Competing economic activities

Most of the people within the study area are predominantly farmers and so most especially during the rainy season when farming is intense, it is always difficult to get them to participate in CHPS activities as they are always busy on their farms. The following are extracts from some participants on how farming activities inhibit community participation in the CHPS programme.

*“We find it difficult to participate in activities of the programme during the rainy season due to farming activities. You know, farming is what we do for a living and so during the rainy season we are always busy on our farms. We leave home as early as 5:30am to the farm and we return around 6:00 pm looking tired.”* (Key Informant 89, Wiaga Zone, Female)

*“We are farmers and that is what we do for a living, we do not have any other means of survival and so when it comes to the farming season we are always occupied with farm work such that it becomes difficult to participate in the activities of the CHPS programme.”* (Key Informant 55, Sandema Zone, Male)

*“When there is a programme or when we schedule a programme during the rainy season, most of the community members go to the bush to farm [...] so when it is in the rainy season around June, July and August there about farming affects most of our community programmes*.”(Key Informant 80 Siniensi Zone, Female)

### Lack of sense of ownership by distant beneficiaries

The health committee members, volunteers and mother-to-mother support group members who hail from the surrounding communities, usually refuse to attend meetings or participate in the activities of the programme because the facility is not located in their communities. Beneficiaries of the programme from the distant communities, therefore, exhibit lack of sense of ownership of the CHPS facilities.

*“Some of our colleagues who come from the surrounding communities are always reluctant to attend meetings and participate in the activities of the programme and when you enquire from them why they are not participating in the programme, they will tell you that, but the facility is yours and not ours so go ahead, the day that we will also get ours, we will participate.”* (Key Informant 36, Kadema Zone, Male)

*“When you call for meetings, some of the health committee members, volunteers and mother-to-mother support group members who are living in the nearby communities will say they are not part of that CHPS compound or they are not part of that community so they would not come, and really, some will not even come there to participate in any* activity in that facility and at the end of the day, it affects the system” (Key Informant 71, Siniensi Zone, Male)

### External contracting of the construction of CHPS facilities

Awarding the construction of CHPS facilities to external contractors by the District Assembly made communities unable to effectively participate in the construction of the CHPS facilities. The respondents reported that the external contractors often rely on their own building materials and labour force outside the community.

*“Hmmm my brother, we could not participate effectively in the construction of the CHPS compound because the compound was given out by the DCE (District Chief Executive) to a contractor who brought in his own building materials and labour force to construct the compound.”* (Key Informant 12, Chuchuliga Zone,Male)

*“Even though we were informed about the construction of the CHPS compound by our MP (Member of Parliament), the contractor when he came to put up the compound did not engage our services throughout the construction process. He single-handedly executed the project with his own resources.”* (Key Informant 66, Sandema Zone,Male)

### Illiteracy

According to key informants, most community members have not had the benefit of formal education and this affects their ability to comprehend many of the health issues that are being discussed. As a result, community members who have been appointed as health volunteers are unable to discharge their responsibilities effectively as health advocates. This, they argued adversely affects community participation in the CHPS programme

*“Most of the community members including myself have not been to school before. This has affected our ability to understand most of the health issues that we are supposed to be educating the community on as health volunteers.”* (*Key Informant 70, Female, Sandema Zone*)

*“I am the chairman of the health committee in this CHPS zone and one of the challenges my colleagues and I are facing here is our inability to read and write in English and so we are unable to appreciate many of the health problems facing us. This is affecting our ability to effectively take part in decision making and also to spread the gospel of health to our people.”* (Key Informant 96, Male, Wiaga Zone)*.*

## Discussion

Our study established that, public education on the CHPS initiative creates awareness of the communities about the CHPS programme and enhances their understanding of their expected roles in the planning and implementation of the programme. This implies that, increasing awareness of community members about the programme and their expected role makes the communities feel they are part of the programme. Such feelings by the beneficiary communities as having a role to play in the planning and implementation of the programme, eliminates apathy and the sense of exclusion by enabling the communities to effectively participate in the analysis of their health needs. In doing so, community participation is enhanced as presented by Rifkin et al. [19]. However, most community members particularly during the rainy season are always busy on their farms and therefore are unable to effectively participate in the analysis of their needs at such times. In addition, most of them have not had the benefit of formal education and thus cannot read and write with little or no ability to research especially into issues of health. This has also affected their ability to effectively analyze their own health needs paving way for technocrats and health professionals to continue to dictate to beneficiary communities about their health needs. This implies that community level literacy interventions are essential to promote effective community participation in PHC.

Our findings also illustrate that, the capacity of the communities to mobilize material resources towards the construction and maintenance of CHPS facilities or compounds promotes community participation in the programme. Locally mobilised material resources play a key role in the successful implementation of the CHPS programme [28]. The ability of stakeholders to contribute these material resources erases the syndrome of inferiority complex among community members, and bolsters their sense of ownership and interest in the planning, implementation and sustainability of the programme [7, 29, 30]. However, external contracting of the construction of CHPS compounds by government officials diminishes the role of beneficiary communities in the mobilization of resources towards the construction of their own CHPS facilities as required by the CHPS policy [16]. For this reason, if the construction of these health facilities is externally driven, it stifles local initiative, which may in the long run, affect the sustainability of the programme. It is, however, important to acknowledge that given the high levels of poverty in the study communities, it is often difficult for the communities to raise sufficient resources on their own for the construction of the CHPS facilities without external intervention. The study findings, therefore, implies that external interventions in the construction of CHPS facilities should focus on providing communities with material and financial resources so that, based on their own local technology, labour and leadership, they can champion the construction of their own CHPS facilities [16]. This will enhance the sense of ownership of the locally constructed CHPS facilities among community members and promote the culture of maintenance and sustainability of the operations of the facilities.

The exercise of strong leadership by the key stakeholders—the chiefs, assembly persons and “*magazias*”—to mobilize the communities to contribute resources for the construction of the CHPS compounds ignites high levels of community participation in the planning and implementation of the CHPS initiative. These key stakeholders who are also gatekeepers of the communities provide strategic direction in the pursuit of health services planning and management. This reinforces the notion of existing literature that, it takes active and dynamic leadership to rally community members to embark on self-help initiatives [31, 32]. Instead of relying on politicians and local government officials to lead the processes for the construction and management of CHIPS facilities, the study finding implies that, there is the need to build the capacity of community level leadership to effectively provide oversight over the implementation of various components of the CHPS programme.

The willingness of communities to avail themselves to support the CHPS initiative without being paid or rewarded promotes community participation in the planning and implementation of the initiative. The CHPS initiative, with the exception of the nurses, makes no provision for rewarding community members who constitute the auxiliary staff and whose effort is so crucial for its success [28]. Even though communities are largely willing and volunteering in the CHPS implementation, this goodwill may be short-lived given that volunteers are not incentivized or rewarded in any form. This scenario may compel volunteers to seek opportunities elsewhere to improve their well-being in the face of poverty. It therefore takes sacrifice and passion for the communities to participate in the planning and implementation of the initiative as indicated by existing literature [31, 33]. Sustainable community participation in the programme will, therefore, require local economic empowerment of the volunteers to reduce volunteer attrition through out-migration, and the provision of periodic allowances and other material livelihood support to the volunteers to motivate them and retain their support for the programme.

Finally, the study also demonstrated that, community participation in the CHPS programme is enhanced when the communities hold the belief that the programme will meet their health needs and aspirations. However, the lack of sense of ownership by distant communities that fall within the catchment area of the facility erodes the trust that those communities hold about the ability of the programme to meet their health needs and aspirations, thereby breeding apathy and lack of commitment towards the programme. Building enthusiasm among programme beneficiaries from distant communities to support programme implementation may require deliberate consensus building around the siting and naming of the CHPS facilities and the involvement of opinion leaders from these distant communities in the leadership and management of the CHPS facilities.

### Limitations of the study

The study was limited to the Builsa North Municipality and therefore, its findings may not be directly transferable to other districts or municipalities within the Upper East Region and beyond. We, therefore, caution against direct generalization of the reported qualitative findings on the enablers and inhibitors of community participation in the programme beyond the study context. The findings were also limited to the views and experiences of only key stakeholders of the CHPS programme and may not therefore reflect the generality of opinions and experiences of the entire community residents of the study area. As a qualitative study, we could not quantify the levels of community participation in the various dimensions of participation as outline by Rifkin [19]. Future studies should cover a statistically representative sample and employ a quantitative approach to estimate the levels and determinants of the community participation in the various dimensions of the programme to complement the findings from our study.

## Conclusion

The study established that, enabling factors to participation include—public education on the CHPS initiative, capacity to contribute material resources towards the construction of CHPS compounds, strong and effective community leadership, the spirit of volunteerism and trust in the benefits of the CHPS programme. However, volunteer attrition, competing economic activities, lack of sense of ownership by distant beneficiaries, external contracting of CHPS facilities’ construction and illiteracy constituted the inhibiting factors. The study recommends that the government of Ghana should extend livelihood intervention programmes to rural communities to minimize volunteer attrition. The Ghana Health Service should tailor most activities of the CHPS programme towards the dry season to avoid absenteeism by volunteers. The government through the municipal assembly should support beneficiary communities with the needed resources and logistics to construct CHPS facilities communally. The Government of Ghana through the Ministry of Health should incentivize and motivate health volunteers with cash, motorbikes, bicycles, torchlights, raincoats and other essentials to meet both their financial and material demands. Finally, the Government of Ghana should make enough budgetary allocations to the Ministry of Health to be able to extend the CHPS programme to many of the communities.

## Data Availability

The study data is available from the corresponding author upon request.

## Abbreviations

CHPS: Community-based Health Planning and Services
GSS: Ghana Statistical Service
KII: Key Informant Interview
MCE: Municipal Chief Executive
NHRC: Navrongo Health Research Center
PHC: Primary Health Care
WHO: World Health Organization
UNICEF: United Nations Children’s Fund
